# Transient Combination Therapy Targeting the Immune Synapse Abrogates T Cell Responses and Prolongs Allograft Survival in Mice

**DOI:** 10.1371/journal.pone.0069397

**Published:** 2013-07-24

**Authors:** Paul M. Schroder, Mithun Khattar, Ronghai Deng, Aini Xie, Wenhao Chen, Stanislaw M. Stepkowski

**Affiliations:** 1 Department of Medical Microbiology and Immunology, University of Toledo College of Medicine, Toledo, Ohio, United States of America; 2 Organ Transplantation Center, 1^st^ Affiliated Hospital, Sun-Yat Sen University, Guangzhou, China; 3 Division of Diabetes, Endocrinology and Metabolism, Department of Medicine, Baylor College of Medicine, Houston, Texas, United States of America; 4 Department of Cardiovascular Surgery, Union Hospital, Huazhong University of Science and Technology, Wuhan, China; Baylor College of Medicine, United States of America

## Abstract

T cells play a major role in allograft rejection, which occurs after T cell activation by the engagement of several functional molecules to form an immune synapse with alloantigen presenting cells. In this study, the immune synapse was targeted using mAbs directed to the TCR beta-chain (TCRβ) and lymphocyte function-associated antigen−1 (LFA1) to induce long-term allograft survival. Evaluation of antigen-specific T cell responses was performed by adoptively transferring CFSE labeled transgenic OT-II cells into wild-type mice and providing OVA peptide by intravenous injection. Graft survival studies were performed in mice by transplanting BALB/c ear skins onto the flanks of C57BL/6 recipients. The anti-TCRβ plus anti-LFA1 mAb combination (but not either mAb alone) abrogated antigen-specific T cell responses invitro and invivo. Transient combination therapy with these agents resulted in significantly prolonged skin allograft survival in mice (51±10 days; *p*<0.01) when compared to treatment with either anti-TCRβ mAb (24±5 days) or anti-LFA1 mAb (19±3 days) alone or no treatment (10±1 days). When lymphoid tissues from these mice were analyzed at different times post-transplant, only those receiving the combination of anti-TCRβ and anti-LFA1 mAbs demonstrated long-lasting reductions in total T cell numbers, cellular and humoral anti-donor responses, and expression of CD3 on the surface of T cells. These results demonstrate that transient anti-TCRβ and anti-LFA1 mAb combination therapy abrogates antigen-reactive T cell responses with long-lasting effects that significantly prolong allograft survival.

## Introduction

Despite advances made over the past few decades in immunosuppressive protocols for transplantation, acute rejection remains a challenge for transplant recipients. The incidence of acute rejection ranges from 10% in renal transplantation to as high as 55% in lung transplantation [1], [2]. T cells play a major role in acute rejection serving as effectors of cellular rejection and helping B-cells produce antibodies responsible for humoral rejection. For this reason, many induction therapies and immunosuppressive regimens have targeted components of the T cell activation process [3].

T cell activation requires engagement of the TCR complex on the surface of T cells with antigen-loaded MHC on antigen presenting cells (APCs) in the form of an immune synapse. Surface molecules on T cells like the TCR, CD28, and lymphocyte function-associated antigen−1 (LFA1) engage molecules such as peptide-MHC, CD80/86, and intercellular adhesion molecule−1 (ICAM1), respectively, on APCs forming the surface connection between the two cell types [4]. These surface molecules are associated with intracellular molecules such as adaptor proteins, kinases, and cytoskeletal components to propagate the surface signals into the full T cell activation cascade [5]. The immune synapse is also known as the supramolecular activation complex (SMAC) and consists of central (cSMAC) and peripheral (pSMAC) components. The cSMAC is largely composed of the TCR-peptide-MHC and co-stimulatory molecule interactions. The pSMAC is largely composed of interactions between adhesion molecules that stabilize the connection between the T cell and APC [6]. Many of these molecules are being investigated as targets of new immunosuppressive agents [7].

The TCR itself has been targeted in the past using mAbs for immunosuppression in transplant recipients [8]. Our group recently demonstrated the effectiveness of an anti-TCRβ mAb in prolonging cardiac allograft survival in mice [9]; however, its effects in more stringent models of acute rejection such as skin transplantation were limited. Antibodies targeting LFA1 have also been successful in immunosuppressive protocols in several rodent models of transplantation [10]–[12], and they have been investigated in humans for treatment of psoriasis and in renal and islet transplantation [13]–[15]. Herein, we applied a unique method to test the effects of various immunotherapies on antigen-specific T cell responses invivo and in turn identified a potent combination therapy of anti-TCRβ and anti-LFA1 mAbs. We demonstrate the efficacy of this combination therapy in prolonging skin allograft survival and investigate its effects on T cell numbers, cellular and humoral anti-donor responses, and expression of cell surface CD3 (a critical TCR signaling component) that contribute to its efficacy.

## Materials and Methods

### Mice

Wild type C57BL/6J (WT C57BL/6) and WT BALB/cJ (WT BALB/c) mice were purchased from the Jackson Laboratory (Bar Harbor, ME). B6.129S7-Rag1^tm1Mom^Tg(TcraTcrb)425Cbn (Rag1^−/−^OT-II) and B6.129S7-Rag1^tm1Mom^Tg(TcraTcrb)1100Mjb (Rag1^−/−^OT-I) mice were obtained from Taconic Farms, Inc. (Hudson, NY).

### Ethics Statement

Animal work was performed in accordance with the Guide for the Care and Use of Laboratory Animals of the National Research Council. Animals were maintained at the University of Toledo Health Science Campus specific pathogen-free animal facility according to institutional guidelines under a protocol approved by the University of Toledo Health Science Campus Institutional Animal Care and Use Committee (Protocol number 105921). All surgery was performed using a mixture of Xylazine plus Ketamine for general anesthesia with subcutaneous injections of Buprenorphine provided for general analgesia, and all efforts were made to minimize suffering of the animals. Animals that showed signs of infection, loss of skin grafts, or excessive bleeding during or after procedures were excluded from the experimental groups and were euthanized by placement in a CO_2_ chamber until cessation of breathing followed by cervical dislocation.

### Reagents

All fluorescence-conjugated mAbs were purchased from either BD Biosciences (San Jose, CA) or eBioscience (San Diego, CA). Purified anti-LFA1 (M17/4) mAb, anti-TCRβ (H57–597) mAb, anti-IL2 (JES6–1) mAb and CTLA4I g fusion protein were obtained from Bio-X-Cell (West Lebanon, NH). Recombinant mouse IL2 was purchased from Peprotech (Rocky Hill, NJ), the OVA_323–339_ peptide (ISQAVHAAHAEINEAGR) was obtained from GenScript USA (Piscataway, NJ) and Ohio Peptide (Powell, OH), and OVA_257–264_ peptide (SIINFEKL) was obtained from Ohio Peptide (Powell, OH). Carboxyfluorescein diacetate succinimidyl ester (CFSE) was obtained from Invitrogen (Carlsbad, CA). ^3^H-thymidine was purchased from Perkin Elmer (Waltham, MA).

### Adoptive Transfer and Stimulation of OT-II Cells

Prior to adoptive transfer, WT C57BL/6 recipient mice were either left untreated or administered the treatments indicated in the text. The IL2/anti-IL2 mAb complex (IL2Cx) treatment was prepared by mixing 2.5 µg IL2 with 25 µg anti-IL2 mAb in sterile PBS and incubating for 30 min at room temperature; the solution was given daily by intra-peritoneal (i.p.) injection on days −6 through −1 before adoptive transfer of OT-II cells. All other treatments were administered by i.p. injection 2 h before adoptive transfer; these included: 250 µg CTLA4Ig, 20 µg anti-LFA1 mAb, 20 µg anti-TCRβ mAb, or combinations of these treatments.

Splenocytes from Rag1^−/−^OT-II mice were CFSE labeled, and 1×10^6^ of these splenocytes were adoptively transferred by intravenous injection into the WT C57BL/6 recipients described above. Upon adoptive transfer the recipient mice received 5 µg OVA_323–339_ by intravenous injection to stimulate the OT-II cells. After 72 h mice were euthanized by placement in a CO_2_ chamber until cessation of breathing, and cervical dislocation was performed before dissection. Spleen and lymph nodes (brachial and inguinal) of the recipient mice were harvested and cell suspensions were stained with fluorescence-conjugated anti-CD4 (GK1.5; eBioscience) alone for detecting CFSE-labeled donor CD4^+^OT-II cells or with anti-CD25 (PC61.5; eBioscience) and anti-Foxp3 (FJK−16 s; eBioscience) for determining the frequencies of CFSE^−^ recipient CD4^+^ and CD4^+^CD25^+^Foxp3^+^ regulatory T cells (Tregs). Flow cytometry was carried out using a BD FACS Calibur machine. Histograms were analyzed using the proliferation tool in FlowJo Software to calculate the division index for selected samples.

### OT-II and OT-I cell Cultures

Splenocytes from Rag1^−/−^OT-II or Rag1^−/−^OT-I mice were CFSE labeled and cultured (0.5×10^6^ cells/well) in a 96 well round-bottom culture plate with stimulation and treatments as indicated in the text. Three days later, the cells were harvested and stained with fluorescence-conjugated anti-CD4 (for OT-II cells) or anti-CD8 (Ly−2; eBioscience; for OT-I cells) for flow cytometry.

### Skin Transplantation and Treatment Regimens

Skin transplants were performed as described previously [9], [16]. Briefly, WT C57BL/6 recipients (8–12 weeks old) and WT BALB/c donors (8–12 weeks old) were anesthetized with a combination of 10 mg/kg Xylazine and 100 mg/kg Ketamine administered by i.p. injection and 0.05 mg/kg Buprenorphine was administered by subcutaneous injection for analgesia. Ear skin (1.0 cm^2^) from the WT BALB/c donor mouse was grafted onto the flank of the WT C57BL/6 recipient. The graft was covered with a sterile bandage, which was removed on day 7 post-transplant. Recipients received either no treatment, 20 µg anti-TCRβ (H57–597), 100 µg anti-LFA1 (M17/4), or a combination of 20 µg anti-TCRβ and 100 µg anti-LFA1. All treatments were diluted in sterile PBS and delivered by i.p. injection on days 0, 1, 3, 7, and 11 post-transplant.

### Cell Subset Analysis by Flow Cytometry

Spleen and lymph nodes (brachial and inguinal) were harvested from skin transplant recipients at days 12, 30, and 60 post-transplant. Cell suspensions were prepared and stained with fluorescence-conjugated anti-CD4, anti-CD8, and anti-CD3ε (17A2; BD Biosciences) for analysis of CD4^+^ and CD8^+^ T cell numbers and CD3 expression or stained with anti-CD4, anti-CD25, and anti-Foxp3 for analysis of Tregs by flow cytometry.

### Mixed Lymphocyte Reaction (MLR)

Splenocytes from skin graft recipient mice and naïve WT C57BL/6 mice were used as responders, while irradiated splenocytes from either WT BALB/c or WT C57BL/6 mice were used as stimulators. Responder cells (0.3×10^6^) were cultured with 0.3×10^6^ irradiated (15 Gy) stimulator cells in a 96-well culture plate for 72 h. Cell proliferation was determined by addition of 1 µCi/well ^3^H-thymidine and measurement of incorporated radioactivity during the final 18 h of culture using a microplate scintillation counter (Packard, Ramsey, MN).

### Detection of Anti-donor IgG in Serum

Sera samples were harvested from the inferior vena cava of anesthetized skin transplant recipients at days 12, 30, and 60 post-transplant as indicated in the text, and were analyzed for the presence of anti-donor (BALB/c) antibody levels using a previously described method [17]. Briefly, WT BALB/c splenocytes were incubated with Fc blocking anti-mouse CD16/CD32 antibody (2.4G2; BD Biosciences) for 25 min at 4°C. Next, cells were washed with 2% fetal bovine serum (FBS) in PBS followed by incubation with 1∶10 dilutions of serum harvested from recipients for 45 min at 4°C. The cells were washed with 2% FBS in PBS and stained with fluorescence conjugated anti-mouse IgG (11–4011–85; eBioscience) for 20 min at 4°C. The cells were washed with 2% FBS a final time and then analyzed by flow cytometry to determine levels of IgG bound to the cells.

### Statistical Analysis

Statistical significance in the graft survival data was determined using the Mann-Whitney test. All other statistical analyses were performed using an unpaired Student’s t-test to determine statistical significance at the levels indicated in the text.

## Results

### Identification of an induction therapy that abrogates antigen specific T cell responses

**Figure 1:**
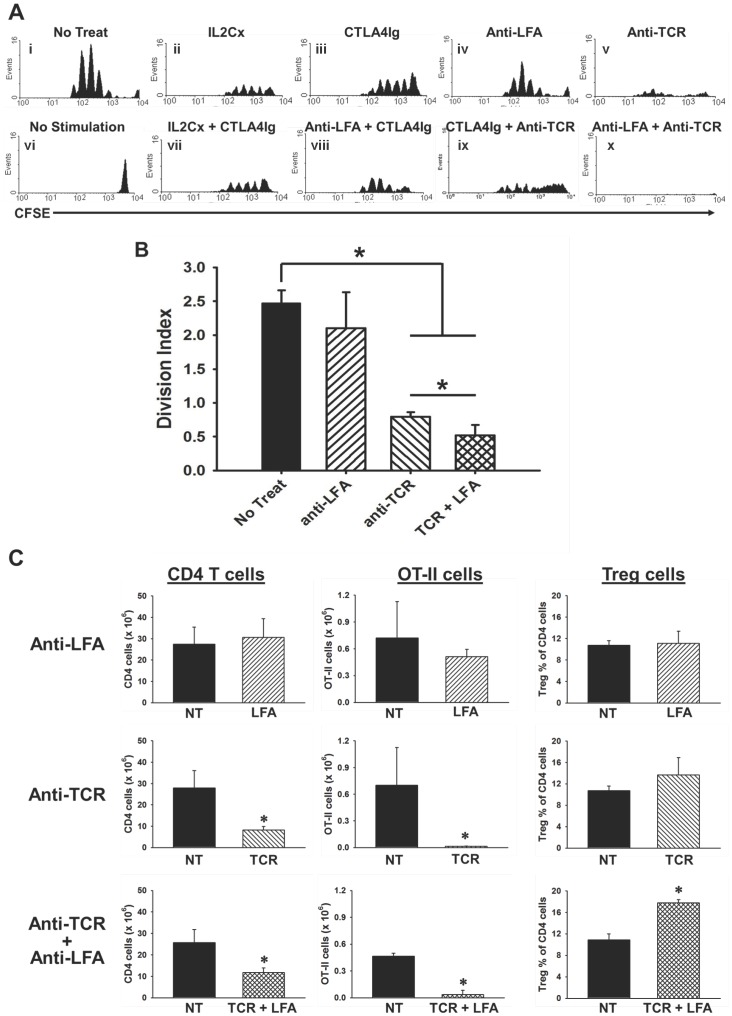
A combination of anti-TCRβ mAb and anti-LFA1 mAb inhibits an antigen-specific T cell response invivo. (**A**) A total of 1×10^6^ CFSE labeled splenocytes from a Rag1^−/−^OT-II mouse were adoptively transferred into C57BL/6 recipients. The recipients were provided with 5 µg OVA_323–339_ to stimulate the OT-II cells (except the No Stimulation group). Histograms show the CFSE^+^ population from 1×10^6^ total events collected from the spleens of recipients who received either (**i**) no treatment (No Treat), (**ii**) 5 µg IL2 and 25 µg anti-IL2 mAb complex treatment (IL2Cx), (**iii**) 250 µg CTLA4Ig, **(**i**v**) 20 µg anti-LFA1 mAb (Anti-LFA), (**v**) 20 µg anti-TCRβ mAb (Anti-TCR), (**vi**) no stimulation, or (**vii–x**) combinations of the above listed treatments as indicated. Each panel is a representative histogram from one of at least three recipients per treatment group. (**B**) Histograms from select groups in A were analyzed using FlowJo Software’s proliferation tool to calculate the division index, which is plotted in the bar graphs for each of the indicated treatment groups. Each bar represents the mean ± standard deviation obtained from three recipients (* indicates *p*<0.05). (**C**) Bar graphs show the total CD4^+^ T cell numbers in spleens (left column), OT-II cell numbers in spleens (middle column), and Treg percentage within the CD4^+^ T cell population in lymph nodes (right column) from the adoptive transfer recipients described above. Each bar represents the mean ± standard deviation obtained from three mice (solid black bars labeled NT represent recipients receiving no treatment while patterned bars represent recipients who received the indicated treatments; * indicates *p*<0.05).

In order to identify a potent induction therapy that could selectively inhibit T cell responses, we developed a unique invivo approach using adoptively transferred CFSE labeled OT-II transgenic (Tg) T cells to evaluate the effects of different immune interventions on the antigen-reactive T cells as well as on the non-reactive CD4^+^ T cells in the WT C57BL/6 recipients (Figure 1A). The first immune intervention tested was the IL2/anti-IL2 mAb complex (IL2Cx) regimen capable of expanding Tregs as described previously [18], [19]. Although the IL2Cx immunotherapy did enhance the Treg population in both spleen and lymph node (Figure S1A), it had only a modest effect on suppressing the OT-II Tg T cell responses (Figure 1Aii). Similarly, treatment with the CTLA4Ig fusion protein, which has been used to prevent allograft rejection [20], [21], resulted in a moderate reduction of the T cell response to antigen stimulation at the indicated dose (Figure 1Aiii). Another immune intervention tested was a mAb targeting the adhesion molecule LFA1, which has been used to treat both autoimmune conditions and allo-rejection [22], [23]. The anti-LFA1 mAb therapy also showed its potential to reduce the population of antigen-responding T cells and shift the distribution of responding T cells toward earlier generations of dividing cells at the indicated dose (Figure 1Aiv). Furthermore, combinations of these therapies showed a modest enhancement in the effectiveness of either of the therapies alone in suppressing the OT-II Tg T cell response invivo (Figure 1Avii–viii).

Recently, our lab demonstrated the efficacy of a mAb therapy targeting the TCR β-chain for prolonging heart allograft survival in mice [9]. Herein, we tested the ability of this anti-TCRβ mAb to suppress the antigen specific OT-II Tg T cell response invivo and showed that it was the most effective in reducing the population of antigen-responding T cells out of all of the monotherapies tested (Figure 1Av). We sought to enhance the effectiveness of the anti-TCRβ mAb by combining it with either CTLA4Ig or anti-LFA1 mAb. While the combination with CTLA4Ig showed similar results to those of anti-TCRβ mAb alone (Figure 1Aix), the combination with anti-LFA1 mAb showed a complete abrogation of the antigen-specific OT-II Tg T cell response invivo (Figure 1Ax). In order to quantify the ability of the anti-TCRβ and anti-LFA1 mAbs combination treatment to suppress the antigen-specific OT-II T cell response, the division index was calculated by FlowJo software (Figure 1B). The division index of the anti-TCRβ plus anti-LFA1 combination was significantly lower than that of either of the two treatments alone.

In order to demonstrate the selectivity of this depletion effect, total CFSE^−^ recipient CD4^+^ T cell numbers and total transferred CFSE^+^ OT-II Tg T cell numbers were compared in the adoptive transfer mice who had received either no treatment or treatment with anti-LFA1 and anti-TCRβ mAbs either as monotherapy or in combination (Figure 1C). There was no significant reduction in the total number of CD4^+^ T cells or in the number of OVA-reactive OT-II Tg T cells in mice treated with anti-LFA1 mAb alone (Figure 1C; top panels). While there was a 70% reduction in the total CD4^+^ T cell numbers in the anti-TCRβ mAb treated mice compared to the untreated mice, there was a greater than 90% reduction in the number of OVA-reactive OT-II Tg T cells in these mice compared to the untreated mice (Figure 1C; middle panels). In mice treated with the combination of anti-TCRβ and anti-LFA1 mAbs, only a 55% reduction was observed in the total number of CD4^+^ T cells, and a greater than 90% reduction in total OT-II Tg T cell numbers was observed compared to the untreated mice (Figure 1C; bottom panels). The combination of the two mAbs also resulted in a significant increase in the percentage of Tregs within the CD4^+^ T cell population (Figure 1C; far right panels and Figure S1B). Together these results show that engagement of T cell synapse components with anti-TCRβ and anti-LFA1 mAbs together resulted in potent abrogation of an antigen specific T cell response, modest reduction of the total CD4^+^ T cell population, and enrichment of Tregs within the CD4^+^ T cell population.

### Anti-TCRβ plus anti-LFA1 mAb Combination Therapy Inhibits T Cell Responses in Vitro

**Figure 2:**
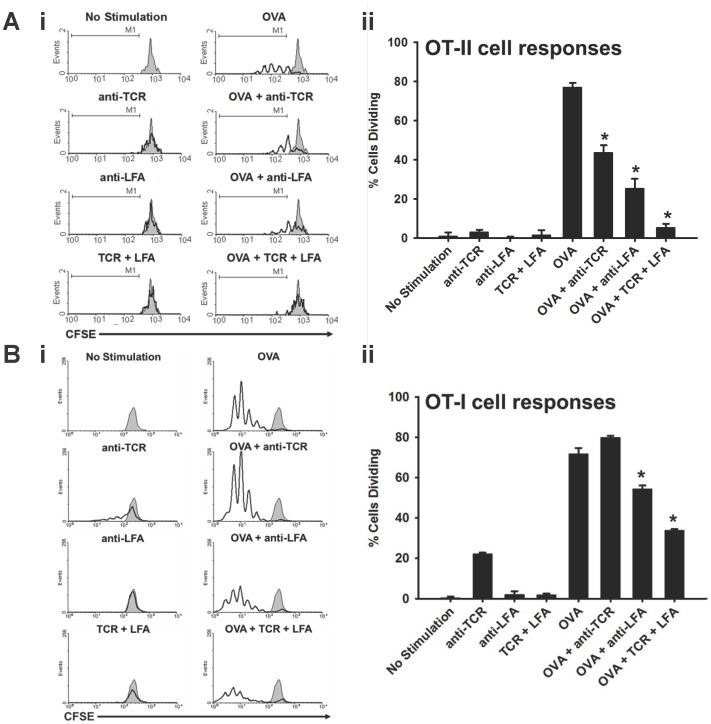
The combination of anti-TCRβ mAb with anti-LFA1 mAb inhibits T cell responses in vitro. (**A)** (**i**) CFSE dilution analysis of labeled OT-II cells cultured with no stimulation (gray shaded histogram), with 5 µg/mL of the indicated mAbs alone (left column; overlay solid black line histograms), or with 5 µg/mL of OVA_323–339_ alone or in the presence of 5 µg/mL of the indicated mAbs (right column; overlay solid black line histograms). (**ii**) Bar graphs show the percentage of dividing cells within the OT-II cell cultures. Each bar represents the mean ± standard deviation of three replicate wells that are representative of two independent experiments (* indicates *p*<0.005 compared to OVA). (**B**) (**i**) CFSE dilution analysis of labeled OT-I cells cultured with no stimulation (gray shaded histogram), with 5 µg/mL of the indicated mAbs alone (left column; overlay solid black line histograms), or with 5 µg/mL of OVA_257–264_ alone or in the presence of 5 µg/mL of the indicated mAbs (right column; overlay solid black line histograms). (**ii**) Bar graphs show the percentage of dividing cells within the OT-I cell cultures. Each bar represents the mean ± standard deviation of three replicate wells that are representative of two independent experiments (* indicates *p*<0.005 compared to OVA).

The following experiments were performed to confirm that the two mAb treatments work by abrogating the T cell response rather than by simply affecting migration or depletion of the T cells. Figure 2A shows the CFSE dilution analysis of the living OT-II Tg T cells from the cultures. While the OT-II Tg T cells cultured without stimulation did not result in significant proliferation (Figure 2Ai; left panels), stimulation with OVA_323–339_ peptide resulted in significant proliferation in the cultures (Figure 2Ai; top right panel). Addition of anti-TCRβ mAb or anti-LFA1 mAb alone to these stimulated cultures reduced proliferation by 43% and 67%, respectively, in the cultures and the combination of the two mAbs resulted in 93% reduction in OT-II Tg T cell proliferation (Figure 2Ai; right panels and Figure 2Aii). Similarly, Figure 2B shows the CFSE dilution analysis of the living OT-I Tg T cells in culture. In the absence of OVA_257–264_ peptide stimulation, the anti-TCRβ mAb in culture was able to stimulate the proliferation of about 20% of the cultured OT-I cells (Figure 2Bi; left panels). While anti-TCRβ mAb alone was unable to decrease the proliferation of OT-I cells in response to OVA_257–264_ peptide stimulation, anti-LFA alone and in combination with anti-TCRβ mAb were able to significantly decrease proliferation of OT-I cells in response to OVA_257–264_ peptide stimulation (Figure 2Bi; right panels and Figure 2Bii). These results show that while either mAb alone can reduce the numbers and generations of dividing T cells upon antigen stimulation, combination of the two mAbs results in nearly complete abrogation of CD4^+^ T cell proliferation and was the most effective method in reducing proliferation of CD8^+^ T cells invitro.

### Anti-LFA1 plus anti-TCRβ mAb Combination Therapy Prolongs Allograft Survival

**Figure 3:**
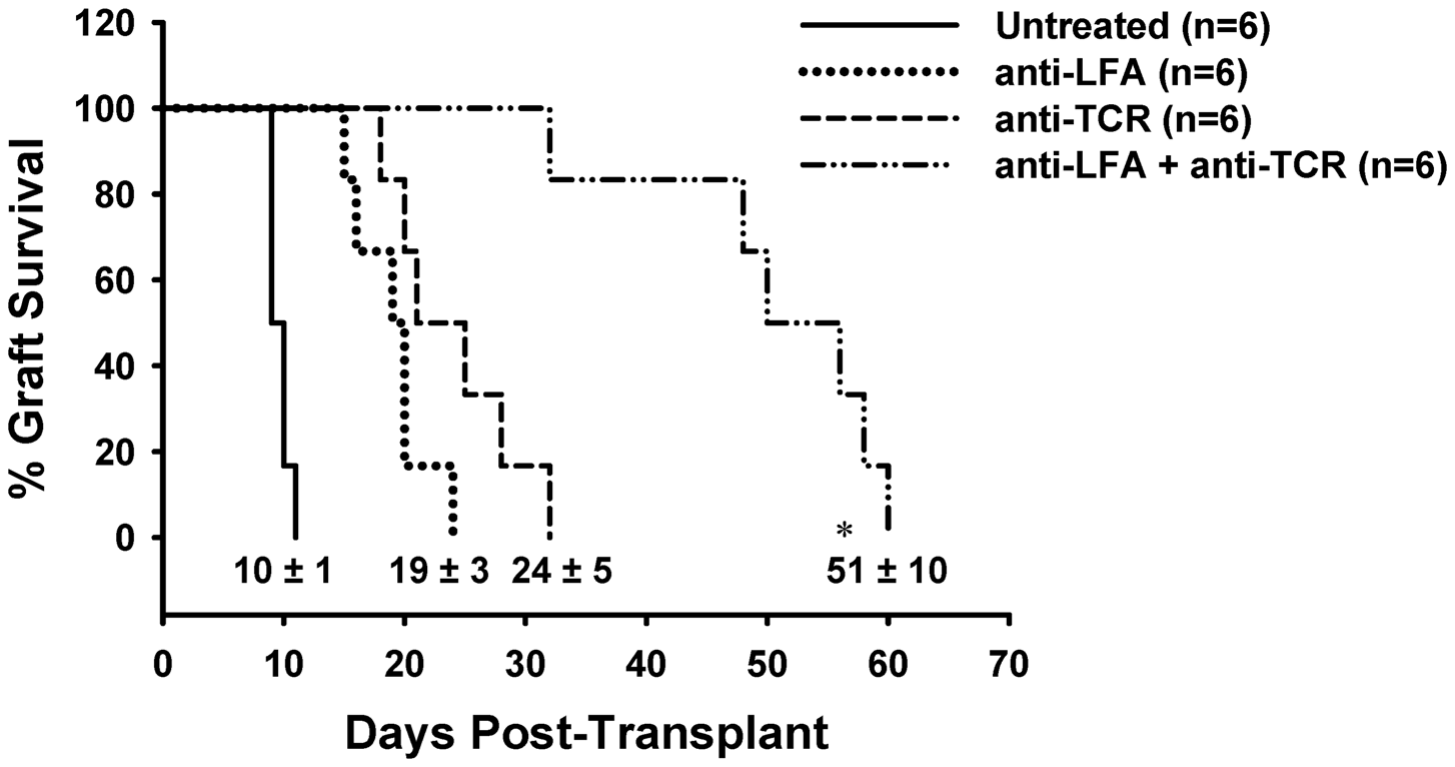
Significant prolongation of allograft survival by the combination therapy of anti-TCRβ and anti-LFA1 mAbs. The plot shows the percent survival of BALB/c skin grafts transplanted onto C57BL/6 recipients either left untreated (solid line) or treated with anti-LFA1 mAb (dotted line), anti-TCRβ mAb (dashed line), or a combination of the two (dashed-dotted line). Numbers indicate the mean survival time of the six mice in each of the treatment groups plus or minus the standard deviation (* indicates *p*<0.01 compared to all other groups).

We then tested whether this combination approach could improve skin allograft survival. The OT-II Tg T-cell adoptive transfer method was used to select an optimal dose of 100 µg anti-LFA1 mAb for use in skin transplantation (Figure S2). WT C57BL/6 recipients of WT BALB/c skin grafts that were left untreated had a mean survival time (MST) of 10±1 days (Figure 3; solid line). Skin allograft survival was prolonged when either anti-LFA1 mAb or anti-TCRβ mAb was administered to the recipients (MST of 19±3 days or 24±5 days, respectively). However, when the combination of these mAbs was used the MST was significantly increased to 51±10 days compared to each monotherapy (Figure 3; dashed-dotted line). Thus, transient treatment with a combination of anti-TCRβ and anti-LFA1 mAbs results in significant prolongation of skin allograft survival.

### Long-term Reduction in T Cell Numbers Following Anti-TCRβ Plus anti-LFA1 mAb Treatment

**Figure 4:**
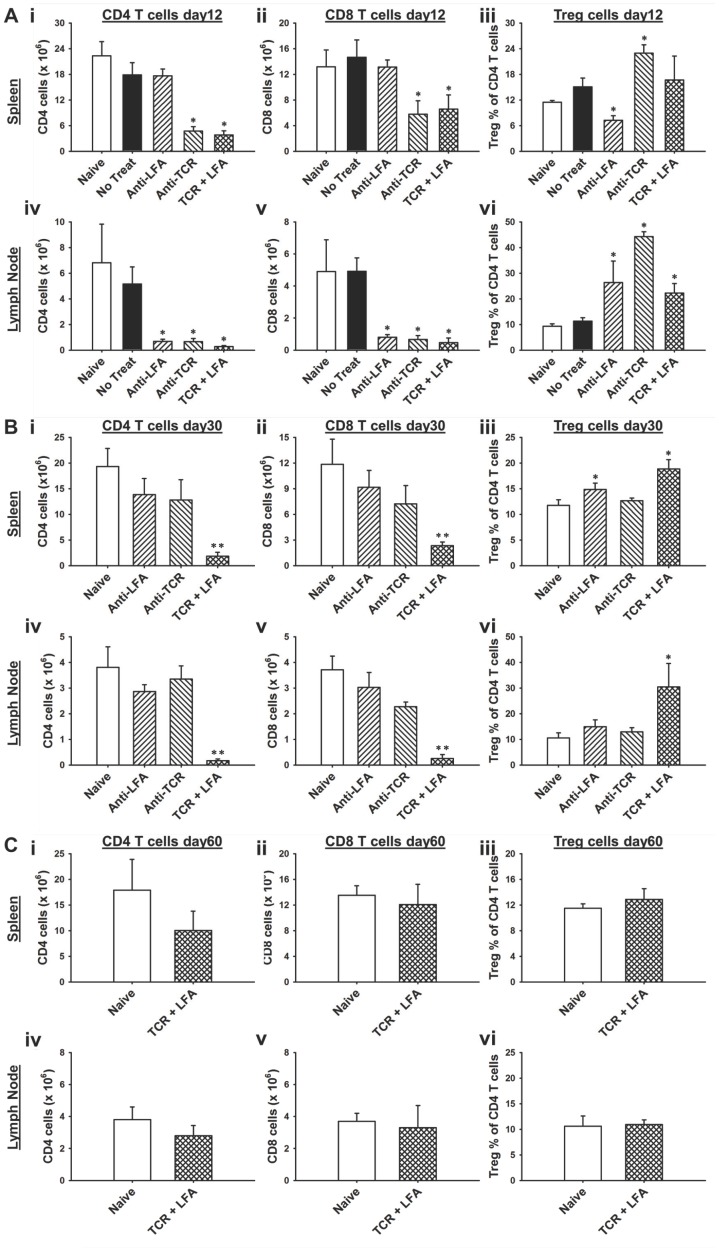
Anti-TCRβ plus anti-LFA1 therapy leads to prolonged depletion of T cells with enhancement of Tregs. (**A**) Lymphoid tissues were harvested at day 12 post-transplant from either naive C57BL/6 mice or C57BL/6 recipients of BALB/c skin allografts administered either no treatment, anti-LFA1 mAb, anti-TCRβ mAb, or the combination therapy. Bar graphs show total CD4 T cell numbers, total CD8 T cell numbers and the Treg percentage within the CD4 T cell population in spleen (**i**, **ii** and **iii**, respectively) and lymph node (**iv**, **v** and **vi**, respectively). Bars show the mean ± standard deviation of values obtained from three mice per group; * indicates *p*<0.05. (**B**) Lymphoid tissues were harvested at day 30 post-transplant from either naive C57BL/6 mice or C57BL/6 recipients of BALB/c skin allografts administered either anti-LFA1 mAb, anti-TCRβ mAb, or the combination therapy. Bar graphs show total CD4 T cell numbers, total CD8 T cell numbers and the Treg percentage within the CD4 T cell population in spleen (**i**, **ii** and **iii**, respectively) and lymph node (**iv**, **v** and **vi**, respectively). Bars show the mean ± standard deviation of values obtained from three mice per group; ** indicates *p*<0.005; * indicates *p*<0.05. (**C**) Lymphoid tissues were harvested at day 60 post-transplant from either naive C57BL/6 mice or C57BL/6 recipients of BALB/c skin allografts administered the combination therapy. Bar graphs show total CD4 T cell numbers, total CD8 T cell numbers and the Treg percentage within the CD4 T cell population in spleen (**i**, **ii** and **iii**, respectively) and lymph node (**iv**, **v** and **vi**, respectively). Bars show the mean ± standard deviation of values obtained from three mice per group.

In order to understand the effects of anti-TCRβ and anti-LFA1 mAbs on T cell populations, lymphoid tissues were harvested from skin allograft recipients at different times post-transplant. At 12 days post-transplant, there were significant reductions in CD4^+^ and CD8^+^ T cell numbers in the spleens of recipients who had received the anti-TCRβ mAb and the anti-TCRβ plus anti-LFA1 mAb treatments compared to untreated recipients (Figure 4Ai–ii). In lymph nodes (LN) at 12 days (Figure 4Aiv–v), there were significant reductions in CD4^+^ and CD8^+^ T cells in all three treatment groups compared to the untreated group. The percentage of Tregs within the CD4^+^ T cell population at day 12 was significantly enhanced in all treatment groups in LN (Figure 4Avi); however, only the anti-TCRβ mAb therapy showed an enhanced Treg population in spleen (Figure 4Aiii).

Analysis of spleen (Figure 4Bi–ii) and LN (Figure 4Biv–v) T cell populations at 30 days post-transplant revealed that T cell numbers had recovered in the anti-LFA1 mAb alone and anti-TCRβ mAb alone treated recipients, while T cell numbers in the combination therapy recipients remained significantly lower. In addition, the Treg percentage within the CD4^+^ T cell population was significantly higher in both spleen and LN in the combination therapy group (Figure 4Biii,vi). However, at 60 days post-transplant, the CD4^+^, CD8^+^, and Treg populations were restored to levels similar to naive mice in both the spleen (Figure 4Ci–iii) and LN (Figure 4Civ–vi) of the combination therapy recipients. Thus, the combination treatment led to prolonged reduction in CD4^+^ and CD8^+^ T cells with enhancement of the Treg population in skin allograft recipients compared to either of the treatments alone.

### Anti-TCRβ plus anti-LFA1 mAb Therapy Results in Prolonged Reduction in Cellular and Humoral anti-donor Responses

**Figure 5:**
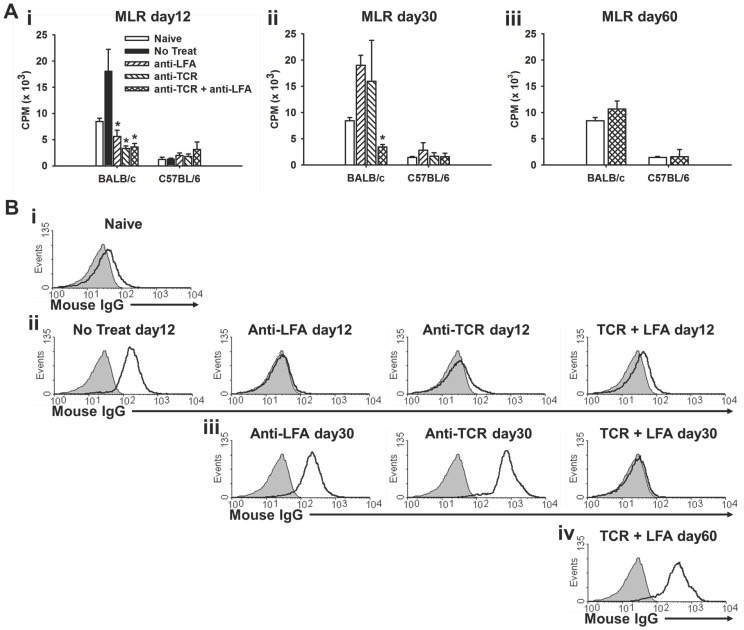
Long-lasting diminution of anti-donor responses in recipients treated with combination anti-TCRβ plus anti-LFA1 mAbs. (**A**) Bar graphs show the average incorporation of ^3^H-thymidine in the mixed lymphocyte reaction cultures of splenocytes harvested at post-transplant day (**i**) 12 (* indicates *p*<0.05 compared to No Treat), (**ii**) 30 (* indicates *p*<0.05 compared to Naive), or (**iii**) 60 from either naive mice or from skin allograft recipients who received the indicated therapies. Each bar represents the mean ± standard deviation of values obtained from splenocyte cultures from three mice. (B) Serum samples were obtained from either (**i**) naive mice or from recipients of skin allografts at post-transplant day (**ii**) 12, (**iii**) 30 or (**iv**) 60 who had received the indicated therapies. The overlay black solid line histograms show the amount of IgG bound to BALB/c splenocytes incubated in the presence of the indicated serum samples while the gray shaded histograms show the no serum control results. Each histogram is one representative sample from three mice in each group.

To examine the alloimmune responses in the skin graft recipients, MLR cultures and anti-donor antibody levels in serum were tested at different times post-transplant. When stimulated with BALB/c antigens in the MLR at 12 days post-transplant, cells from recipients who had received no treatment showed significant ^3^H-thymidine incorporation. None of the recipient mice who had received treatment with either anti-TCRβ mAb, anti-LFA1 mAb, or both showed a substantial response in the MLR culture at day 12 (Figure 5Ai). However, significant ^3^H-thymidine incorporation at 30 days was seen in the anti-TCRβ mAb and anti-LFA1 mAb monotherapy groups, which was not observed in the combination treatment group (Figure 5Aii). The cellular response to donor tissue was recovered in the combination treatment group by 60 days (Figure 5Aiii).

Donor-specific antibodies in sera of the recipients were also evaluated at different days post-transplant. At day 12, only untreated recipients showed significant amounts of anti-donor IgG in serum (Figure 5Bi–ii). By day 30, recipients who had received either mAb therapy alone showed significant anti-donor IgG in their serum, but combination therapy recipients showed no detectable anti-donor IgG (Figure 5Biii). However, by day 60, significant levels of anti-donor IgG were detected in sera of combination therapy recipients (Figure 5Biv). Collectively, these results show that there were long-lasting diminished cellular and humoral anti-donor responses in skin transplant recipients receiving the combination therapy compared to those that had received either mAb alone or no treatment.

### Anti-TCRβ and anti-LFA1 mAb Combination Results in Long-lasting Reduction in the Amount of the T Cell Surface Signaling Component CD3

**Figure 6:**
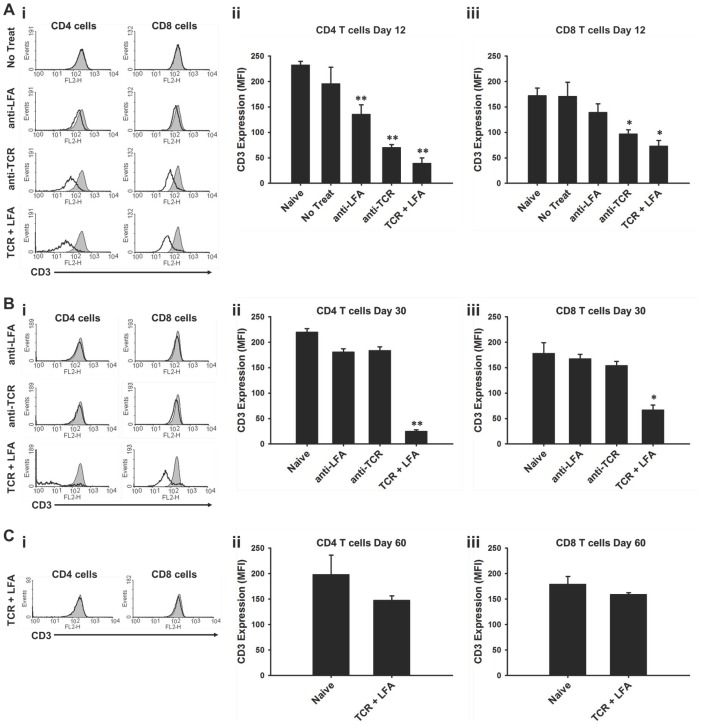
Anti-TCRβ plus anti-LFA1 mAb treatment results in long-term reduction of CD3 expression on T cells. (**A**) Cells from lymph nodes were harvested from either naive mice or skin allograft recipients treated with the indicated therapies for analysis of CD3 expression on T cells at day 12 post-transplant. (**i**) Overlay black solid line histograms show the CD3 expression on the surface of CD4 (left panels) and CD8 (right panels) T cells from one representative treated allograft recipient while gray shaded histograms show the result from a naive mouse. (**ii** & **iii**) Bar graphs show the average mean fluorescent intensity (MFI) for CD3 expression on the surface of CD4 T cells and CD8 T cells, respectively (** indicates *p*<0.005; * indicates *p*<0.05). Each bar represents the mean ± standard deviation of values obtained from three mice. (**B**) Cells from lymph nodes were harvested as in A at day 30 post-transplant. (**i**) Overlay black solid line histograms show the CD3 expression on the surface of CD4 (left panels) and CD8 (right panels) T cells from one representative treated allograft recipient while gray shaded histograms show the result from a naive mouse. (**ii** & **iii**) Bar graphs show the average MFI for CD3 expression on the surface of CD4 T cells and CD8 T cells, respectively (** indicates *p*<0.005; * indicates *p*<0.05). Each bar represents the mean ± standard deviation of values obtained from three mice. (**C**) Cells from lymph nodes were harvested as in A at day 60 post-transplant. (**i**) Overlay black solid line histograms show the CD3 expression on the surface of CD4 (left) and CD8 (right) T cells from one representative treated allograft recipient while gray shaded histograms show the result from a naive mouse. (**ii** & **iii**) Bar graphs show the average MFI for CD3 expression on the surface of CD4 T cells and CD8 T cells, respectively. Each bar represents the mean ± standard deviation of values obtained from three mice.

Since this combination therapy targeted two components of the immune synapse, we were interested in monitoring surface expression of other important signaling components after treatment. Figure 6 shows expression of CD3 on the surface of T cells harvested from skin transplant recipients at different times post-transplant. The amount of CD3 on the surface of CD4^+^ T cells was significantly reduced in all of the treated recipient groups at day 12 post-transplant, while the untreated recipients showed CD3 expression similar to that of naive mice (Figure 6Ai–ii). However, only the anti-TCRβ mAb monotherapy and combination therapy groups showed significant reductions in CD3 expression on the surface of CD8^+^ T cells at day 12 (Figure 6Aiii). At day 30, CD3 expression on the surface of both CD4^+^ and CD8^+^ T cells in recipients treated with either mAb alone had recovered, while CD3 levels on the surface of T cells from combination therapy recipients remained significantly lower (Figure 6Bi–iii). By day 60, CD3 expression on CD4^+^ and CD8^+^ T cells had fully recovered in the combination therapy recipients (Figure 6Ci–iii). These results show that while anti-TCRβ mAb or anti-LFA1 mAb alone can alter CD3 expression on T cells in the short-term, long-lasting reduction in CD3 levels on the surface of T cells was only observed when the combination therapy was administered.

## Discussion

We have demonstrated a unique in vivo model for evaluating the effectiveness of immune interventions in suppressing antigen-specific T cell responses and used this model to select an effective combination therapy for use in preventing allograft rejection. There have been many adoptive transfer models used to investigate the antigen-specific T cell responses in vivo including models of transplantation, infection, and autoimmunity [24]–[26]. However, our model uses a simplified approach to stimulate adoptively transferred OT-II T cells by injecting the OVA peptide, allowing us to observe and evaluate the response of those antigen-responding CD4^+^ T cells and the recipient’s non-responding T cells to different immune interventions. Analyses of these recipients showed that treatment with the anti-TCRβ/anti-LFA1 mAbs resulted in nearly complete and more selective depletion of the antigen responding CD4^+^ T cells compared to the non-responding population of CD4^+^ T cells. It is known that both CD4^+^ and CD8^+^ T cells play important roles in mediating the rejection of allografts; however, we focused on the antigen-specific CD4^+^ T cell responses in our model due to the critical role that CD4^+^ T cells play in activation and direction of many other cell types including CD8^+^ T cells during the immune response against allografts [27].

The anti-LFA1 mAb has been used as an immune intervention alone and in combination in several models of allograft transplantation in rodents including skin, heart, and islet transplants [28]–[30]. Since the anti-LFA1 mAb prevents the interaction of LFA1 with ICAM1, its mechanism of action is generally thought to be two-fold. First, anti-LFA1 mAb blocks the migration of activated T cells to sites of inflammation [31]. A second mechanism involves disruption of the contact between T cells and APCs to prevent full activation of lymphocytes [31]–[33]. Our results are consistent with both of these mechanisms. When mouse recipients of skin allografts were treated with anti-LFA1 alone, there was a reduction in CD4^+^ T cells in LN but not in the spleens of these mice, suggesting that anti-LFA1 mAb affected the distribution of cells between these two organ types in vivo. Our results from the antigen specific OT-II invivo response in the presence of a single injection of anti-LFA1 alone did not result in an increase in the Treg population after three days. However, a five dose treatment with anti-LFA1 in the skin allograft recipients did result in a significant increase in the Treg population in the lymph nodes of these mice at day 12 post-transplantation, which is similar to other reports using anti-LFA1 therapy in mice and humans [34], [15]. In addition, our invitro culture of the OT-II T cells in the presence of either anti-LFA1 mAb alone or in combination with anti-TCRβ mAb demonstrated that these treatments abrogate T cell responses in the absence of a migration effect.

Our lab recently demonstrated that the anti-TCRβ mAb is effective in prolonging cardiac allograft survival in mice and showed that the antibody therapy results in a depletion of the antigen-reactive T cells with enhancement of the Treg population [9]. Indeed, our results are consistent with these findings in skin transplant recipients who received anti-TCRβ mAb therapy alone or in combination with anti-LFA1 mAb. In particular, anti-TCRβ mAb treatment reduced the numbers of CD4^+^ and CD8^+^ T cells with enhancement of the Treg population, which was long-lasting in recipients treated with the combination therapy. This significant and long-term depletion of T cells is a likely explanation for the reduced proliferation seen in the MLR cultures and the low levels of anti-donor antibody detected in the serum of recipients treated with the combination therapy. However, we cannot rule out the intrinsic effects of anti-TCRβ/anti-LFA1 mAbs on the remaining T cell populations [35], [36]. Our own data show that these mAbs alone and in combination reduced the expression of the CD3ε surface signaling molecule on both CD4^+^ and CD8^+^ T cells, which may have impaired the ability of the remaining T cells to mount an effective immune response until the surface CD3 expression was recovered. These findings are consistent with previous reports of receptor internalization during engagement with specific mAbs as well as the activation threshold of T cells being set, in part, by the number of receptors expressed on the cell surface [37]–[39].

The main focus of this study was to identify a combined therapy that most potently abrogates an acute allo-response. Transient anti-TCRβ mAb treatment alone induces long-term survival (>100 days) of heart [9] and islet (unpublished observation) allografts even when the frequencies of immune cells have completely recovered. Therefore, to efficiently detect the combination effects of anti-TCRβ and anti-LFA1 mAbs, we chose the more stringent skin transplantation model in which each treatment alone exhibits only modest effects. Indeed, the combination therapy significantly prolonged skin graft survival when compared to each treatment alone, but immune cell recovery in skin graft recipients was always associated with the recurrence of allograft rejection. An ideal therapy would allow for full recovery of immune cell frequency and function against non-transplant antigens with permanent allograft acceptance. In our ongoing projects, we compare various transplantation models to reveal the mechanisms that mediate the rejection or acceptance of allografts by the recovered immune cells.

Both anti-TCRβ mAb and anti-LFA1 mAb therapies have shown effectiveness as part of immunosuppressive regimens in the clinic [40]–[43]. In addition, both were used as successful induction therapies in transplant patients [14], [44]. However, anti-TCR mAbs have been largely neglected in clinical use, and anti-LFA1 mAb was recently removed from the market in 2009 for its risk of reactivation of latent JC virus infection [45], [46]. Recently, there has been renewed interest in the use of anti-LFA1 mAb in short-term settings or in combinations that can reduce its dosing regimen [47]. Our results indicate that the combination of these two agents provides a significant benefit for prolonging skin allograft survival in mice. The short-term therapy used in this study demonstrates the feasibility of using such a combination as an effective induction therapy that can decrease the doses of these mAbs and help enhance effectiveness while limiting the potential side effects of long-term and high dose use of these therapies.

## Supporting Information

Figure S1 **Enhancement of the Treg cell population in mice treated with IL2Cx and anti-TCRβ/anti-LFA1 combination therapy during OT-II adoptive transfer.**
(DOC)

Figure S2 **Titration of anti-LFA1 mAb dose for use in skin transplantation.**
(DOCX)
