# Supplementary material for: Transient Combination Therapy Targeting the Immune Synapse Abrogates T Cell Responses and Prolongs Allograft Survival in Mice
Source: PLoS One. 2013 Jul 24;8(7):e69397. doi: 10.1371/journal.pone.0069397 (PMC3722282; doi:10.1371/journal.pone.0069397)
Supplement: Figure S1 — Enhancement of the Treg cell population in mice treated with IL2Cx and anti-TCRβ/anti-LFA1 combination therapy during OT-II adoptive transfer. (DOC) [file pone.0069397.s001.doc]

**SUPPORTING INFORMATION**

**Figure S1. *Enhancement of the Treg cell population in mice treated with IL2Cx and anti-TCRβ/anti-LFA1 combination therapy during OT-II adoptive transfer.*** **(A)** Representative dot plots showing CD25 and Foxp3 expression. Dot plots are gated on total CD4+ lymphocytes from either spleen (top row) or lymph nodes (bottom row) of C57BL/6 mice either left untreated (No Treat) or treated for 5 days with IL2/anti-IL2 mAb complex (IL2Cx) either before (Day 0) or 3 days after (Day 3) adoptive transfer of CFSE labeled OT-II splenocytes. Numbers indicate the percentage of CD25+Foxp3+ Treg cells within the CD4+ lymphocyte population. **(B)** C57BL/6 mice were either left untreated (No Treat) or treated with the indicated mAbs and adoptively transferred with CFSE-labeled OT-II splenocytes. The Dot plots show CD25 and Foxp3 expression gated on the total CD4+ lymphocytes from either spleen (top row) or lymph nodes (bottom row) of the mice at day 3 after adoptive transfer. Numbers indicate the percentage of CD25+Foxp3+ Treg cells within the CD4+ lymphocyte population.
