# Supplementary material for: Transient Combination Therapy Targeting the Immune Synapse Abrogates T Cell Responses and Prolongs Allograft Survival in Mice
Source: PLoS One. 2013 Jul 24;8(7):e69397. doi: 10.1371/journal.pone.0069397 (PMC3722282; doi:10.1371/journal.pone.0069397)
Supplement: Figure S2 — Titration of anti-LFA1 mAb dose for use in skin transplantation. (DOCX) [file pone.0069397.s002.docx]

**SUPPORTING INFORMATION**

**Figure S2. *Titration of anti-LFA1 mAb dose for use in skin transplantation.*** A total of 1 x 10^6^ CFSE labeled splenocytes from a Rag1^−/−^OT-II mouse were adoptively transferred into C57BL/6 recipients. The recipients were provided with 5 μg OVA_323-339_ to stimulate the OT-II cells. Histograms show the CFSE^+^ population from 1 x 10^6^ total events collected from the spleens of recipients who received either no treatment or one injection of anti-LFA1 mAb at the indicated doses.
